# The Impact of an Intervention Taught by Trained Teachers on Childhood Overweight

**DOI:** 10.3390/ijerph9041355

**Published:** 2012-04-16

**Authors:** Rafaela Rosário, Bruno Oliveira, Ana Araújo, Oscar Lopes, Patrícia Padrão, André Moreira, Vítor Teixeira, Renata Barros, Beatriz Pereira, Pedro Moreira

**Affiliations:** 1 School of Nursing, University of Minho, Largo do Paço, 4704-553 Braga, Portugal; 2 Research Centre in Child Studies, School of Education, University of Minho, Campus de Gualtar, 4710-057 Braga, Portugal; Email: anam_araujo@iol.pt (A.A.); beatriz@ie.uminho.pt (B.P.); 3 Faculty of Nutrition and Food Sciences, University of Porto, Rua Roberto Frias, 4200-465 Porto, Portugal; Email: bmpmo@fcna.up.pt (B.O.); patriciapadrao@fcna.up.pt (P.P.); vhugoteixeira@fcna.up.pt (V.T.); renatabarros@fcna.up.pt (R.B.); pedromoreira@fcna.up.pt (P.M.); 4 Sports’Medical Center, Tempo Livre, Alameda Cidade de Lisboa, Creixomil, 4835-037 Guimarães, Portugal; Email: olopes@tempolivre.pt; 5 Faculty of Medicine, University of Porto, Al. Prof. Hernâni Monteiro, 4200-319 Porto, Portugal; Email: andremoreira@med.up.pt; 6 Research Centre in Physical Activity, Health and Leisure, Universiy of Porto, Rua Dr. Plácido Costa, 91, 4200-450 Porto, Portugal

**Keywords:** BMI z-score, children, obesity, overweight, trained teachers, health promotion

## Abstract

The purpose of this study was to assess the effects of a six-months’ nutrition program, delivered and taught by classroom teachers with in-service nutrition training, on the prevention of overweight and obesity among children in grades 1 to 4. In this randomized trial, four hundred and sixty four children from seven elementary schools were allocated to a nutrition educational program delivered by their own teachers. Intervened teachers had 12 sessions of three hours each with the researchers throughout six months, according to the topics nutrition and healthy eating, the importance of drinking water and healthy cooking activities. After each session, teachers were encouraged to develop activities in class focused on the learned topics. Sociodemographic, anthropometric, dietary, and physical activity assessments were performed at baseline and at the end of the intervention. In the intervention group the increase in Body Mass Index (BMI) z-score was significantly lower than in the control group (*p* = 0.009); fewer proportion of children became overweight in the intervened group compared with the control (5.6% *vs.* 18.4%; *p* = 0.037). Our study provides further support to decrease the overweight epidemic, involving classroom teachers in a training program and making them dedicated interventionists.

## 1. Introduction

The prevalence of obesity continues to increase [1] and is a growing concern in Portugal and around the World. Overweight and obesity have serious implications in child’s health as they have been associated with asthma, type II diabetes, cardiovascular disorders, hepatic and vesicular diseases, stigma and other psychological problems, sleep apnea, arthritis, stroke, certain type of cancers and quality of life [2]. As the process of treatment is complex, discussion about how to prevent obesity is currently high on public health agenda and effective health promotion remains a key strategy [3]. 

Although the picture of how to intervene is far from complete, guidance developed by effective programs have led to an increase in obesity prevention research, but so far only yielded “best practice” recommendations. In addition, most studies have been developed in the United States, raising questions about their applicability in European countries given the different school system and school nutrition conditions, as well as differences in eating habits or obesity rates [3,4]. As a result, there is a need to develop and implement effective intervention programs and policies in Europe aiming to improve children’s lifestyles.

Interventions in childhood are considered to have larger effects than in older ages because children are more sensitive to outside influences and may consider interventions particularly attractive [5]. Moreover, eating and physical activity behaviors learned during childhood may persist throughout adulthood [5,6]. The potential setting of the interventions is also an important aspect of overweight and obesity prevention programs. The use of the community structures, such as schools, reduces the barriers of implementation and focus in a natural setting for children [5].

Until now, the role of teachers in the delivery features of the interventions and its impact on child’s body composition is unclear [7,8]. In addition, four studies examine the effects of educational programs on the anthropometry of children [9,10,11,12] and few meet the real knowledge of teachers and children’s needs. Theoretically, teachers are not able to devote as much time and energy to providing interventions as dedicated interventionists because teachers have classroom responsibilities that take precedence [8]. To the best of our knowledge, this is the first study that inserts the intervention program to prevent childhood overweight in the progression of teaching career. 

The purpose of the present research was to assess the impact of a six months nutrition program, delivered and taught by classroom teachers with in-service nutrition training, on the prevention of overweight and obesity among children in grades 1 to 4. 

## 2. Experimental Section

### 2.1. Participants

**Figure 1:**
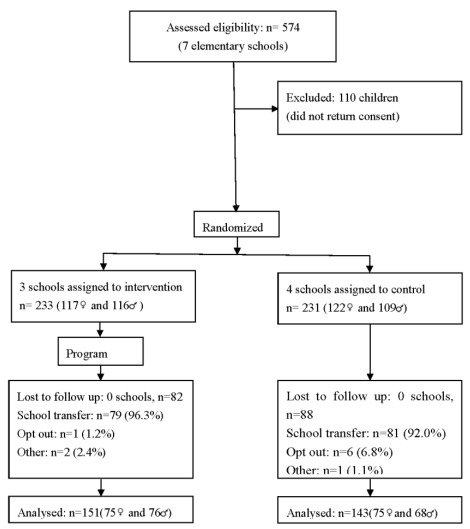
Flow of participants through each stage of the program.

During 2007/2008 seven out of eighty public elementary schools were randomly selected and invited to participate in this study. The number of schools involved was according to constraints of personnel for the assessment and implementation of the program. Schools were the unit of randomization and three were assigned into intervention, and four into control group (Figure 1). The distance between schools was of 5 Km or less and all of them were considered urban. Data was collected prior to the program and immediately after. Prior to participation on data collection, parents provided informed consent, according with the ethical standards laid down in the Declaration of Helsinki, and children provided oral assent. The study was approved by the schools where it was carried out and by the Portuguese Data Protection Authority (CNPD-Comissão Nacional de Protecção de Dados, process number 7613/2008). In addition the protocol for this study was registered in the clinical trials registry clinicaltrials.gov, NCT01397123.

The flow of the subjects during the study is presented in Figure 1. From the 574 children who were invited to participate, 464 (239 female, ages ranging from 6 to 12 years old) agreed and returned the written consent forms filled by their parents. Of these, 233 (50.2%) were allocated to the intervention group, and 231 (49.8%) to the control group. Follow up assessment was available for 63.4% of children, 143 (61.9%) in the control and 151 (64.8%) in the intervention groups. 

BMI and major sociodemographic characteristics did not differ significantly between the children who participated in the baseline and those not included in the final assessment.

Attrition rates did not differ between intervention and control group (35.2% and 38.1%, respectively). Major reasons for nonparticipation were school transfer (94.1%), parent refusal (4.1%) and absence from school (1.8%). Children and outcomes assessors were blinded to group assignment. A total of 257 parents of the children involved in the study provided data at baseline and 203 (79.0%) at post-intervention. 

### 2.2. Overview of the Program

Teachers from intervention schools were invited and agreed to participate in the program conducted between October 2008 and March 2009; fifteen teachers were involved. This program was based on the Health Promotion Model [13] and the social cognitive theory [14] and aimed to promote healthier active lifestyles by encouraging children to be more active and ensure better food selection. 

The Health Promotion Model argues that actions and the health promotion behavior are a corollary of personal characteristics, prior experiences, perceived benefits and barriers for action as well as perceived self-efficacy [13]. This program was aware of these influences on children behavior and focused on a positive vision of health. Likewise the social cognitive theory, the program enhanced cognitive and behavioral skills by enabling children to make changes in their own behavior and to employ new choices effectively [14].

In Portugal, in-service training is considered the basis for the efficacy of the Education system. Teachers have the right and the obligation to develop their training through an accumulation of credits. Therefore, the research team proposed the accreditation of the training sessions developed with the teachers to the Minister of Education, Scientific-Pedagogic Council for In-service Training (Conselho Científico Pedagógico da Formação Contínua, Ministério da Educação). It was approved in the form of a “training workshop” with 72 h duration, distributed between active learning strategies (36 h with the researchers) and the delivery of the learnt contents to the children (36 h). Thus, the program was implemented over two terms with teachers training delivered by researchers and intervention delivered by trained teachers to children. 

Teachers of the intervention group had 12 sessions of three hours each with the researchers during six months, which included the following contents: session 1, how to promote health and prevent disease, lifestyle determinants of health, obesity—definitions and descriptions of the problem, risk factors and health problems; session 2, key concepts in food and nutrition; sessions 3 and 4, dietary guidelines (the Portuguese Food Wheel), healthy eating advice for children, covering the five main food groups, and interventions to help children and their families to consume healthy foods and plan well-balanced meals and snacks; session 5, teach children about the importance of water, and teaching strategies to replace consumption of sugar-sweetened beverages with water; sessions 6 and 7, appropriate physical activity levels and healthy eating behaviours such increasing fruit and vegetable intake and decreasing energy-dense micronutrient-poor foods; session 8, teaching strategies and learning theory in the classroom; session 9, strategies to reduce screen exposure time; session 10, global assessment of the training program; sessions 11 and 12, healthy cooking and strategies to get children and their families involved in healthy cooking.

After each session, teachers delivered the learnt contents and developed creative and engaging classroom activities about the addressed topic. All the questions that arose during the implementation of classroom activities were addressed and resolved shortly with the researchers. Teachers were allowed to develop and refine the activities and the learning strategies that were proposed by the researchers. At the end of this period, the teachers delivered a critical report of activities focused on the intervention, evaluated by the research team. The autonomy of teachers in the training process was preserved and the researchers had a serious investment on professionalism, pragmatism and policy [15]. Recognizing that it was not possible to complete a structured assessment of all the information and materials delivered in all of the classes, researchers also performed an informal assessment (based on face-to-face interviews by asking questions and clarifying unresolved issues) on each teacher.

The implementation of the program occurred as planned. All the children of the intervention schools had contact with trained teachers. Teachers taught the components of the program as prescribed and the researchers were always available to answer any question. In addition, teachers reported they were enthusiastic about the training, and had a total attendance in the sessions with the researchers. 

### 2.3. Assessments

In each school, previously trained persons performed anthropometric evaluation, using standardized procedures [16]. Anthropometric measurements were performed in children with light indoor clothing and barefooted. Weight was measured in an electronic scale, with an error of ±100 g (Seca, Model 703, Germany), and height was measured using a stadiometer, with the head in the Frankfort plane. BMI was computed as mass, kg/height m^2^. The prevalence of underweight, normal weight, overweight and obesity was calculated according to the International Obesity Task Force (IOTF) criteria [17]. A z-score (the number of standard deviations (sd) from the reference population) was calculated for each child using the LMS method and the calculation was determined using the LMS growth add-in for excel [18]. To estimate the magnitude of BMI changes during the study, a new variable was computed from the difference between the post-intervention and baseline BMI z-score. 

Dietary intake was gathered by one day 24-h dietary recall obtained by nutritionists and/or trained interviewers before and after the program. Children did not have prior notification of when the recalls would occur in order to prevent potentially biasing reports and the weekend days were avoided. During the 24-h dietary recall, each child was asked to recall all food and beverages consumed during the past 24 h. Portion sizes of foods and beverages consumed were also estimated, using food models and photos, and other props (cups, glasses, food wrappers or containers) as an aid in determining serving sizes. Daily routines were used as prompts (waking up, going to bed, time between classes, and before or after school) to enhance recall. Energy and nutritional intake were estimated using an adapted Portuguese version of the nutritional analysis software Food Processor Plus (ESHA Research Inc., Salem, OR, USA). 

In order to assess the level of physical activity of children, parents were asked five questions with four answer choices (4-point scale) ranging from 1 to 4 about children’s activity [19]: (a) outside the school does your child take part in organized sport? (b) outside school does your child take part in non-organized sport? (c) outside school, how many times a week does your child take part in sport or physical activity for at least 20 min? (d) outside school hours, how many hours a week does your child usually take part in physical activity so much that gets out of breath or sweat? (e) does your child take part in competitive sport?. This questionnaire was developed by Telama *et al.* [20] and its appliance to Portuguese population has been described previously [19,21]. Overall a maximum of 20 points can be reached. The Physical Activity Index was obtained, splitting the sample into four activity classes: sedentary group [5]; low activity group [6,7,8,9,10]; moderately active group [11,12,13,14,15]; and vigorously active group [16,17,18,19,20], on the basis of their reported physical activity [19,22].

Social, demographic and family characteristics were assessed by questionnaire. The survey sent to parents contained questions about gender and age of children, education of the parents (recorded in five categories: 0, 1–4, 5–9, 10–12, and more than 12 years of formal education). This information was further grouped for analysis into three categories: up to 9 years, 10–12 years, and more than 12 years of education.

### 2.4. Statistical Analyses

Mean and sd were used to describe continuous variables. Student’s *t*-tests, Mann-Whitney U, Kruskall Wallis and Chi-square tests were used to compare several variables grouped by intervention and control school groups and sex. These tests were also conducted to assure comparability of anthropometric measurements between groups at baseline. A 0.05 level of significance was considered. For the purpose of sample size estimation, the primary outcome was a change in BMI-z score between post-intervention and baseline. According to previous studies [23], a sd of BMI z-score near 0.5 and a dropout rate near 30% was estimated. From these assumptions a power analyses was conducted using sample size for each group of 143, obtaining an estimated power of 0.84 for *t*-test.

The effect of the program was evaluated based on changes in anthropometry between baseline and post intervention, comparing intervention to control schools. The tests examining these differences were developed using Generalized Linear Models and took into account the nested nature of the data (children were nested within schools). Hence, the BMI z-score variation was used as dependent variable and the adjustment was made for gender, age, baseline total energy intake, baseline BMI z-score and parents’ education in order to maximize precision. 

Incidence of overweight and obesity after intervention (percentage of children who were not initially overweight or obese but who became overweight or obese), prevalence (percentage of children who were overweight or obese) and remission (percentage of children who were overweight or obese at baseline but were not overweight or obese after intervention) were analyzed. Analyses of overweight and obesity were conducted separately. The prevalence, incidence and remission assessment was conducted by binary outcomes.

The data analysis was performed using SPSS^®^, Version 18.0 (SPSS Inc; Chicago, IL, USA). 

## 3. Results

Table 1 shows the baseline characteristics of the participants. Subjects included 239 girls and 225 boys with a mean (sd) age of 8.3 (1.2) years. The average BMI was 17.9 kg/m^2^ (sd = 2.7, range 11.9 to 26.9 kg/m^2^) and BMI z-score was 0.8 (1.1). Overall, 23.3% of the children were classified as overweight and 9.5% as obese. The large majority of the children (≈65%) were classified in sedentary or low activity group. Mean energy intake was not significantly different between intervention and control schools (respectively, 2091 (684) kcal *versus* 2024 (582) kcal, *p* = 0.257). No significant differences were found for weight, BMI and BMI z-score at the baseline assessment between intervention and control groups.

**Table 1:** Characteristics of the sample at baseline in intervention and control schools.

N	Total	Intervention	Control	p
	464	231	233	
Age (years)	8.3 (1.2)	8.3 (1.2)	8.2 (1.2)	0.846
Boys	225 (48.5)	116 (49.8)	109 (47.2)	
Girls	239 (51.5)	117 (50.2)	122 (52.8)	0.575
**Mother’s education**				
Up to 9 years	244 (64.0)	116 (58.6)	128 (69.9)	
10–12 years	88 (23.1)	52 (26.3)	36 (19.7)	
>12 years	49 (10.6)	30 (15.2)	19 (10.4)	0.021
**Father’s education**				
Up to 9 years	254 (69.0)	122 (62.9)	132 (75.9)	
10–12 years	70 (19.0)	39 (20.1)	31 (17.8)	
>12 years	44 (12.0)	33 (17.0)	11 (6.3)	0.003
Weight (kg)	30.9 (7.4)	30.9 (7.2)	30.9 (7.5)	0.901
Height (cm)	130.5 (7.8)	129.3 (7.8)	131.3 (7.8)	0.046
BMI (kg/m^2^)	17.9 (2.7)	18.1 (2.7)	17.7 (2.8)	0.062

Data presented as mean (sd) for age, anthropometric variables, and *n* (%) for other variables.

There were significant differences between groups with regard to mother and father education (*p* = 0.021 and *p* = 0.003 respectively), and height (*p* = 0.045). There were higher levels of parents education in the intervention group and children in the control group were taller [1.29 (0.06) vs. 1.27 (0.06) m]. To account for these differences at baseline, these variables were controlled for in subsequent analyses.

After intervention (Table 2), the BMI z-score variation (post intervention—baseline) was higher in the control than in the intervention subjects [respectively, mean (se) 0.34 (0.05) *versus* 0.13 (0.04)]. After adjusting for gender, age, baseline total energy intake, baseline BMI z-score and parents’ education, the BMI z-score increased 0.176 units more in the control group than in the intervention group [95% CI = (0.044;0.308), *p* = 0.009], not represented in the table.

**Table 2:** Outcomes at post-intervention.

Measure	Baseline		Post-intervention		*p*
	Control	Intervention	Control	Intervention	
	*n* = 233	*n* = 231	*n* = 143	*n* = 151	
BMI z-score	0.66 (1.12)	0.84 (1.07)	0.92 (1.0)	0.90 (0.97)	0.875
Energy (kcal/day)	2024.2 (581.8)	2091 (683.9)	2475.6 (684.9)	2388.0 (1036.5)	0.399
**IOTF**					
Underweight	10 (2.1)	7 (1.5)	0 (0.0)	2 (0.7)	
Normal	157 (33.8)	138 (29.7)	90 (62.9)	95 (62.9)	
Overweight	41 (8.8)	67 (14.4)	40 (28.0)	44 (29.1)	
Obesity	23 (5.0)	21 (4.5)	13 (9.1)	10 (6.6)	0.610
**Physical Activity Index**					
Sedentary	21 (15.6)	23 (14.0)	6 (7.1)	5 (5.9)	
Low-activity	72 (53.3)	82 (50.0)	48 (56.5)	40 (47.1)	
Moderately activity	35 (25.9)	49 (29.9)	26 (30.6)	30 (35.3)	
Vigorously Activity	7 (5.2)	10 (6.1)	5 (5.9)	10 (11.8)	0.133

Data presented as mean (sd) for BMI z-score, energy and *n* (%) for other variables.

Significantly fewer children in the intervention schools (5.6%) than in the control schools (18.4%) became overweight after the intervention (unadjusted means; Table 3). After controlling for confounders, the predicted odds of incidence was 75% lower for the intervention group (odds ratio [OR]: 0.25; 95% CI: 0.07–0.92; *p* < 0.05). Furthermore, as expected, overweight children with a BMI closer to normal weight status were more likely to have remission of overweight than overweight children with a higher BMI (*p* = 0.018). Similarly, normal weight children on the threshold of being overweight were more likely to suffer of overweight compared with normal weight children with a lower BMI (p = 0.001), not represented in the table. More intervened children had remission of overweight compared to control group (13% and 12.5%, respectively) although non-significantly. After intervention there were no significant differences in the prevalence, incidence or remission of obesity between groups of schools.

**Table 3:** Prevalence, incidence and remission of overweight and obesity after intervention.

Measure	Sample, *n*	Baseline, *n* (%)	Post-intervention, *n* (%)	Unadjusted change (%) ^a^	Adjusted Odds (95% CI)^ b^	*p*
**Overweight Prevalence**						
Control	143	24 (16.8)	40 (28.0)	11.2	1.00	0.880
Intervention	151	46 (30.5)	44 (29.1)	−1.4	1.10 (0.31; 3.97)	
Incidence						
Control	98	-	18 (18.4)	18.4	1.00	0.037
Intervention	89	-	5 (5.6)	5.6	0.25 (0.07; 0.92)	
Remission						
Control	24	-	3 (12.5)	−12.5	1.00	0.835
Intervention	46	-	6 (13.0)	−13.0	1.24 (0.17; 9.31)	
**Obesity Prevalence**						
Control	143	14 (9.8)	13 (9.1)	−0.7	1.00	0.493
Intervention	151	9 (6.0)	10 (6.6)	−0.6	0.42 (0.04; 4.94)	
Incidence						
Control	24	-	4 (16.7)	16.7	1.00	0.979
Intervention	46	-	5 (10.9)	10.9	0.98 (0.16; 5.64)	
Remission						
Control	14	-	5 (35.7)	−35.7	1.00	0.493
Intervention	9	-	4 (44.4)	−44.4	2.36 (0.20; 27.45)	

*N* = 294 (individuals with data at baseline and post-intervention. Sample sizes for prevalence included all 294 participants, whereas sample sizes for incidence and remission were dependent on initial weight status (e.g., incidence of overweight was based only on individuals who were normal weight at baseline, whereas remission of obesity was considered using only individuals who were obese at baseline). - indicates no data available; ^a^ Data are unadjusted percentages; ^b^ Odds were adjusted for gender, age, baseline total energy intake, parents’ education and baseline BMI z-score.

## 4. Discussion

Our study shows that a nutrition program, delivered and taught by in-service teachers trained in nutrition, is effective in lowering the increase in the incidence of overweight among schoolchildren. Intervened children have a significantly lower increase in BMI and a lower proportion of children become overweight. This is particularly important as it provides further evidence to the WHO recommendations for schools to include both dietary and physical activity components in the curriculum taught by trained teachers [24]. 

Our study has some weaknesses that should be mentioned. We did not explore differences between schools selected and not selected given human and materials constraints, although the schools were from the same geographical area and to the best of our knowledge, no data is available reporting sociodemographic and income significant differences between schools from this particular area. In addition, to the best of our knowledge there were no other “anti-obesity” programs going on in intervention and control schools.

It is possible that our sample size was not large enough to detect other significant differences than those reported. Furthermore, we failed to obtain identically equivalent groups after randomization, namely in parents education level and children’s height, mainly because we randomized by school and not by subjects, aiming to avoid cross contamination between intervention and control groups. Nevertheless, these differences were taken in account in all of the statistical models. Also, physical activity levels were obtained upon self-reported data making recall bias and overestimation possible. However, the questionnaire was validated for Portuguese adolescents [19] and we have no reason to assume these biases would affect groups differently. Future studies would be improved by using accelerometry to better classify physical activity.

On the other hand, the present study has important strengths that also should be acknowledged. First, to the best of our knowledge this is the first study that included the program in the progression of teaching career. This probably allowed teachers to increase their motivation in the delivery of the intervention. It would be desirable that other similar programs could be recognized on the career progression of teachers in Portugal and other countries in order to engage and reward teachers for their efforts. Second, the long term teachers’ in-service training and the subsequent network developed between themselves, researchers and children. We know that in Portugal students of education courses do not have health promotion in their training curricula, neither consider its changes towards an increase in health content important [25]. In addition, teachers are described as a group that is not able to devote much time and energy to provide obesity prevention interventions as dedicated interventionists [8]. Being aware of this need and that long-term programs are more effective than those of short duration [8] we promoted a six months duration training program expecting teachers to become also nutrition educators. We believe this period allowed teachers to recognize just how important healthy eating habits and physical activity are. Third, our approach was to standardize recommendations to teachers, allowing them enough flexibility to create interactive interventions and pedagogic instruments to be used with children. The teachers were assessed after their intervention for material and lessons developed, and it was found that the information incorporated in their lessons and/or the quality of teaching materials did not vary between them. This is in line with the development of “scholarship of teaching and learning” [15] and is contrary to previous school-based interventions that have used tight controls to ensure uniform implementation but required frequent staff training and ongoing supports [26]. Fourth, the intervention mapping developed to identify the real needs in knowledge of teachers and children helped to make the translation of objectives to change strategies. Finally, we estimated dietary intake using the 24 h dietary recall method, which is the most commonly used method in Europe [27]. In addition, we assessed the effectiveness of the program using BMI values expressed as z-scores avoiding age and gender effects. It would be desirable that children from the present study could be followed to see whether the results still hold through life cycle, but no resources exist to further support this study.

We observe that most children are in the sedentary or low activity group. These results are in line with previous pediatric studies [28].

We further observe a very high prevalence of overweight and obesity in our subjects, emphasizing the epidemic state of overweight/obesity in Portuguese children and adolescents [29]. Actually, this is also a global problem, as confirmed by other studies where the prevalence of childhood overweight continues to increase [1,30,31]. Therefore, during the last years several programs have been developed aiming to reduce childhood overweight and obesity incidence and prevalence [10,26,32,33,34]. However, the results of these studies also indicate the resiliency of body weight because only few of them significantly reduced average body weight or adiposity in intervention groups of girls or boys [33]. In this context, this study yielded encouraging results as it reflects the positive effect of an educational program on anthropometric status, little seen in Europe [35]. Nevertheless, we should be cautious in the interpretation of our results, given the small sample size and the short follow-up in our study. Similar results as in the present study were found by James *et al.* [10], Borys *et al.* [9] and Panunzio [11]. The intervention duration of the studies that reported effect on anthropometry varied from thirty six weeks [11] to five years [12], although the latter did not have a positive effect on anthropometry [12]. Besides the effect on overweight, the intervention had no effect on the prevalence, incidence and remission of obesity, and further studies should find out probable reasons for inefficacy on obesity. Progression to or remission from the upper end of BMI distribution may be more likely to result from targeted and/or clinic-based programs than from primary prevention such this program. Moreover, the program was tailored to the specific study population and may not be as effective in a different group with different needs. The positive results may reflect in part aspects of the Portuguese educational system and motivation of teachers may be less in countries where the in-service training is not included in the progression of teaching career.

## 5. Conclusions

In conclusion, our program targeting classroom teachers as dedicated interventionists has significant effects on overweight prevention. It is well recognized that public health efforts are crucial to avoid childhood overweight and obesity as well as later problems. Changes in dietary behavior may be brought about, not by direct modification of food habits, but by alteration or manipulation of education and culture [36]. Therefore, we consider that teachers play a key role in successful implementation of healthier lifestyles that have impact on childhood overweight and obesity. 

We believe that future studies with larger samples and longer periods of follow-up should try to replicate these findings to assess if this approach could be disseminated to other school districts. In addition, future directions should center on other aspects like school environment, physical education classes or on the environment beyond the school, such as homes and stores. The already high prevalence of overweight in children with this age and the incidence of new cases of overweight and obesity, even in intervention schools, leads to the need to improve the effect, dose, and range of prevention programs directed to children younger than 6 years.

## References

[1] Ogden C.L., Carroll M.D., Curtin L.R., McDowel M.A., Tabak C.J., Flegal K.M. (2006). Prevalence of overweight and obesity in the United States, 1999–2004. J. Am. Med. Assoc..

[2] Bray G.A. (2004). Medical consequences of obesity. J. Clin. Endocrinol. Metab..

[3] Branca F., Nikogosian H., Lobstein T. (2007). The Challenge of Obesity in the WHO European Region and the Strategies for Response, Summary.

[4] Currie C., Gabhainn S.N., Godeau E., Roberts C., Smith R., Currie D., Picket W., Richter M., Morgan A., Barnekow V. (2008). Inequalities in Young People’s Health: HBSC International Report from the 2008/2006 Survey.

[5] Kumanyika S.K., Kumanyika S.K., Obarzanek E., Stettler N., Bell R., Field A.E., Fortmann S.P., Franklin B.A., Gillman M.W., Lewis C.E. (2008). Population-based prevention of obesity: The need for comprehensive promotion of healthful eating, physical activity, and energy balance: A scientific statement from American Heart Association Council on Epidemiology and Prevention, interdisciplinary committee for prevention (formerly the expert panel on population and prevention science). Circulation.

[6] Whitlock E.P., Williams S.B., Gold R., Smith P.R., Shipman S.A. (2005). Screening and interventions for childhood overweight: A summary of evidence for the US Preventive Services Task Force. Pediatrics.

[7] Sharma M. (2006). International school-based interventions for preventing obesity in children. Obes. Rev..

[8] Stice E., Marti C.N. (2006). A meta-analytic review of obesity prevention programs for children and adolescents: The skinny on interventions that work. Psychol. Bull..

[9] Borys J.M., Lafay L. (2000). Nutritional information for children to modify the food habits of the whole family. Rev. Med. Suisse Romande.

[10] James J., Thomas P., Kerr D. (2004). Preventing childhood obesity by reducing consumption of carbonated drinks: Cluster randomized controlled trial. Br. Med. J..

[11] Panunzio M., Antoniciello A., Pisano A., Dalton S. (2007). Nutrition education intervenion by teachers may promote fruit and vegetable consumption in italian students. Nutr. Res..

[12] Angelico F., Del Ben M., Fabiani L., Lentini P., Pannozzo F., Urbinati G.C., Ricci G. (1991). Management of childhood obesity through a school-based programme of general health and nutrition education. Public Health.

[13] Pender N.J. (1996). Health Promotion in Nursing Practice.

[14] Bandura A. (1986). Social Foundations of Thought and Action: A Social Cognitive Theory.

[15] Shulman L. (2000). From minsk to pinsk: Why a scholarship of teaching and learning?. J. Scholarsh. Teach. Learn..

[16] World Health Orgnization Expert Committee (1995). Physical Status: The Use and Interpretation of Anthropometry.

[17] Cole T.J., Bellizi M.C., Flegal K.M., Dietz W.H. (2000). Establishing a standard definition for child overweight and obesity worldwide: International survey. Br. Med. J..

[18] Pan H., Cole T. (2009). lmsGrowth, a Microsoft Excel add-in to access growth references based on the LMS method (Version 2.68). http://www.healthforallchildren.co.uk/.

[19] Mota J., Esculcas C. (2002). Leisure-time physical activity behavior: Structured and unstructured choices according to sex, age, and level of physical activity. Int. J. Behav. Med..

[20] Telama R., Yang X., Laakso L., Viikari J. (1997). Physical activity in childhood and adolescence as predictor of physical activity in young adulthood. Am. J. Prev. Med..

[21] Ledent M., Cloes M., Piéron M. (1997). Les jeunes, leur activité physique et leurs perceptions de la santé, de la forme, des capacités athlétiques et de l’apparence [The youth people. Their physical activity and their perceptions of health, fitness, performance and shape]. Sport.

[22] Raitakari O.T., Porkka K.V., Taimela S., Telama R., Rasanen L., Viikari J.S. (1994). Effects of persistent physical activity and inactivity on coronary risk factors in children and young adults. The Cardiovascular Risk in Young Finns Study. Am. J. Epidemiol..

[23] Pedrosa C., Oliveira B.M., Albuquerque I., Simoes-Pereira C., Vaz-de-Almeida M.D., Correia F. (2011). Metabolic syndrome, adipokines and ghrelin in overweight and obese schoolchildren: Results of a 1-year lifestyle intervention programme. Eur. J. Pediatr..

[24] Anderson J., Parker W., Steyn N.P., Grimsrud A., Kolbe-Alexander T., Lambert E.V., Mciza Z., Armstrong T., Candeias V., Bruin T., Xuereb G. (2009). Interventions on Diet and Physical Activity: What Works, Summary Report.

[25] Precioso J. (2004). Educação para a saúde na Universidade: Um estudo realizado em alunos da Universidade do Minho. Rev. Electrón. Enseñ. Cienc..

[26] Hoelscher D., Feldman H.A., Johnson C.C., Lytle L.A., Osganian S.K., Parcel G.S., Kelder S.H., Stone E.J., Nader P.R. (2004). School-based health education programs can be maintained over time: Results from the CATCH institutionalization study. Prev. Med..

[27] European Food Safety Authority (2009). General principles for the collection of national food consumption data in the view of a pan-European dietary survey. Eur. Food Saf.Auth. J..

[28] Mota J., Fidalgo F., Silva R., Ribeiro J.C., Santos R., Carvalho J., Santos M.P. (2008). Relationships between physical activity, obesity and meal frequency in adolescents. Ann. Hum. Biol..

[29] Moreira P. (2007). Obesity in Portuguese children and adolescents. J. Public Health.

[30] Liou T.H., Huang Y.C., Chou P. (2009). Prevalence and secular trends in overweight and obese Taiwanese children and adolescents in 1991–2003. Ann. Hum. Biol..

[31] Edwards K.L., Clarke G.P., Ransley J.K., Cade J.E. (2010). Serial cross-sectional analysis of prevalence of overweight and obese children between 1998 and 2003 in Leeds, UK, using routinely measured data. Public Health Nutr..

[32] Eisenmann J.C., Gentile D.A., Welk G.J., Callahan R., Strickland S., Walsh M., Walsh D.A. (2008). Switch: Rationale, design, and implementation of a community, school, and family-based intervention to modify behaviors related to childhood obesity. BMC Public Health.

[33] Foster G.D., O’Keeffe M., Matthews J.N., Mathers J.C., Nelson M., Barton K.L., Wrieden W.L., Adamson A.J. (2008). A policy-based school intervention to prevent overweight and obesity. Pediatrics.

[34] Caballero B., Clay T., Davis S.M., Ethelbah B., Rock B.H., Lohman T., Norman J., Story M., Stone E.J., Stephenson L., Stevens J. (2003). Pathways: A school-based, randomized controlled trial for the prevention of obesity in American Indian schoolchildren. Am. J. Clin. Nutr..

[35] Van Cauwenberghe E., Maes L., Spittaels H., van Lenthe F.J., Brug J., Oppert J.M., De Bourdeaudhuij I. (2010). Effectiveness of school-based interventions in Europe to promote healthy nutrition in children and adolescents: Systematic review of published and “grey” literature. Br. J. Nutr..

[36] Fieldhouse P. (1998). Food and Nutrition: Customs and Culture.

